# Proximity Labeling in Plants

**DOI:** 10.1146/annurev-arplant-070522-052132

**Published:** 2023-02-28

**Authors:** Shou-Ling Xu, Ruben Shrestha, Sumudu S. Karunadasa, Pei-Qiao Xie

**Affiliations:** 1 Department of Plant Biology, Carnegie Institution for Science, Stanford, California, USA; 2 Carnegie Mass Spectrometry Facility, Carnegie Institution for Science, Stanford, California, USA; 3 Department of Molecular and Cell Biology, University of California, Berkeley, California, USA

**Keywords:** proximity labeling, protein–protein interaction, protein–nucleotide interaction, subcellular proteome, cell type–specific proteome, quantification

## Abstract

Proteins are workhorses in the cell; they form stable and more often dynamic, transient protein–protein interactions, assemblies, and networks and have an intimate interplay with DNA and RNA. These network interactions underlie fundamental biological processes and play essential roles in cellular function. The proximity-dependent biotinylation labeling approach combined with mass spectrometry (PL-MS) has recently emerged as a powerful technique to dissect the complex cellular network at the molecular level. In PL-MS, by fusing a genetically encoded proximity-labeling (PL) enzyme to a protein or a localization signal peptide, the enzyme is targeted to a protein complex of interest or to an organelle, allowing labeling of proximity proteins within a zoom radius. These biotinylated proteins can then be captured by streptavidin beads and identified and quantified by mass spectrometry. Recently engineered PL enzymes such as TurboID have a much-improved enzymatic activity, enabling spatiotemporal mapping with a dramatically increased signal-to-noise ratio. PL-MS has revolutionized the way we perform proteomics by overcoming several hurdles imposed by traditional technology, such as biochemical fractionation and affinity purification mass spectrometry. In this review, we focus on biotin ligase–based PL-MS applications that have been, or are likely to be, adopted by the plant field. We discuss the experimental designs and review the different choices for engineered biotin ligases, enrichment, and quantification strategies. Lastly, we review the validation and discuss future perspectives.

## 1. INTRODUCTION

Plant cells have unique structural and functional features that evolved in response to environmental cues (59). Elucidating the complex cellular networks that underlie plant-specific functions at the molecular level is critical to understand plant growth or development and to dissect genotype-to-phenotype relationships. In particular, knowledge about protein abundance, localization, protein–protein interactions (PPIs), and the intimate interplay between protein and DNA/RNA will shed light on the functional and mechanical understanding of plants’ unique molecular capabilities. However, obtaining such data remains challenging, especially for weak and transient interactions, low-abundant proteins, and hard-to-purify subcellular structures (97). In addition, significant proportions of genes and the proteins they encode in plants are yet to be functionally characterized (98).

**Figure 1:**
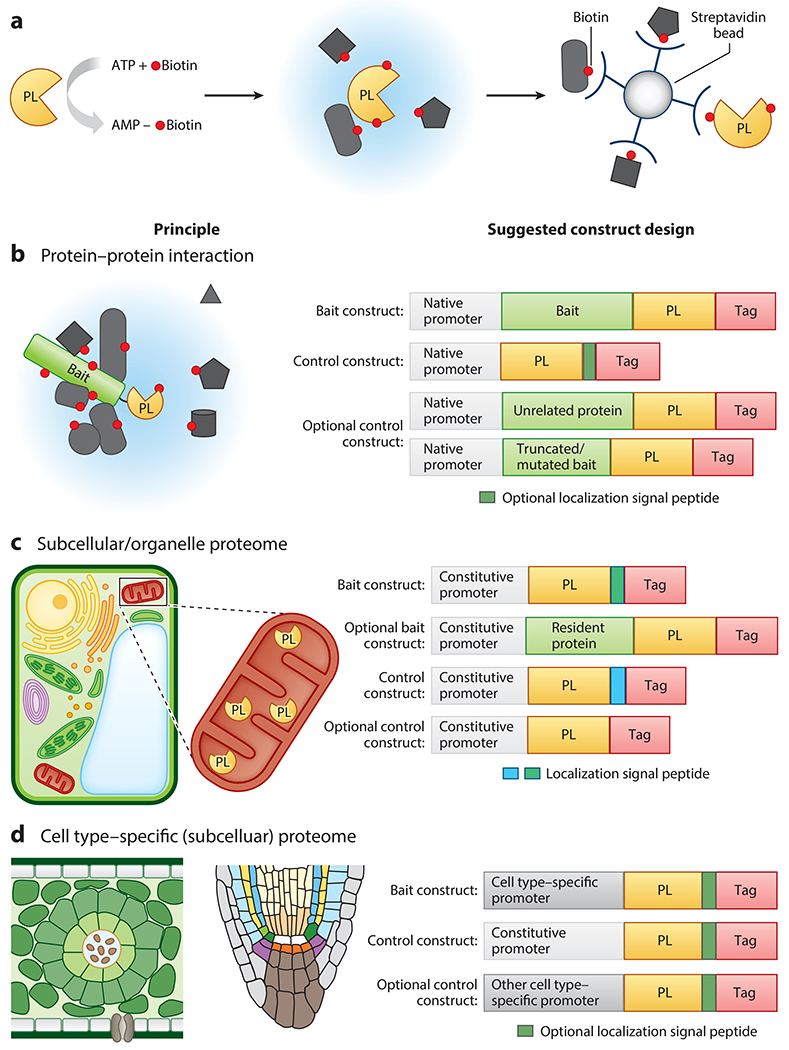
The general principle and application of PL in studying protein–protein interactions, subcellular and organelle proteomes, and cell type–specific proteomes. (*a*) Depiction of biotin ligase PL. The PL enzyme reacts with biotin and ATP to form a diffusible, reactive intermediate biotinoyl-5′-AMP that biotinylates proximal proteins. The blue region denotes the biotinylation radius. Biotinylated proteins are subsequently enriched using streptavidin beads followed by mass spectrometric analysis. (*b–d*) Principle (*left*) and suggested construct designs (*right*) of different types of PL-MS studies. (*b*) Depiction of PL-MS applications used to study protein–protein interactions. The PL enzyme is fused to the bait protein expressed at the endogenous level to target a protein complex. Upon the addition of biotin, the PL enzyme biotinylates proteins in its vicinity. (*c*) Depiction of a PL-MS application used to study subcellular/organelle proteomes. A schematic view of a plant cell is shown with its membrane-bound or membraneless compartments, exemplifying a PL application in the mitochondria matrix. (*d*) Depiction of PL-MS used to study cell type–specific (subcellular) proteomes. Example proteomes include bundle-sheath cells and mesophyll cells in C_4_ plants and *Arabidopsis* root cells in the root tip. Tags (e.g., GFP/Myc/Flag) are often included to visualize protein localization and to assess protein expression levels. The positions of the tag, bait protein, PL enzyme, and localization signal peptide can be rearranged to allow the proper expression and function of constructs in PL. Abbreviations: AMP, adenosine monophosphate; ATP, adenosine triphosphate; GFP, green fluorescent protein; Myc, a peptide tag derived from the c-Myc protein; Flag, a short, hydrophilic protein tag; MS, mass spectrometry; PL, proximity labeling.

Biotin ligase–based proximity-labeling (PL) technology has emerged as a powerful tool for studying complex cellular networks. In PL, a genetically encoded biotin ligase is targeted to a protein complex of interest or to an organelle by fusing to a protein or a localization signal peptide. In the presence of applied biotin, this engineered promiscuous biotin ligase converts biotin into short-lived, diffusible, and activated biotin adenylate intermediates, which are transferred to the ε-amino group of surface-exposed lysine residues of proximal proteins within a 10-nm radius (101) (Figure 1a). The biotinylated proteins are then enriched by streptavidin beads and subsequently identified by mass spectrometry (MS). Multiple versions of biotin ligases have been engineered, such as BioID (BirA*) (55), BioID2, ultraID and microID2 (10, 49, 60), TurboID and miniTurboID (10), and AirID (54). In particular, the advent of TurboID (10) allows for highly efficient labeling in a timescale of minutes and a broader range of working temperatures. These improvements on PL have enabled proteomic mapping in a spatial and temporal manner and extended its use to many more organisms, including plants. Moreover, the recent coupling of PL with clustered regularly interspaced short palindromic repeats (CRISPR) has allowed more biological questions to be answered (78, 133).

PL has several major advantages. First, PL allows the tagging of proteins in a physiological context as the PL enzyme is genetically encoded and expressed in vivo. Maintaining intact protein complexes or organelles during lysis and purification is often challenging when using traditional methods such as affinity purification and biochemical fractionations. PL circumvents this hurdle by tagging both bait and prey proteins or by tagging constituent proteins within an organelle with biotin, then capturing them using the high-affinity streptavidin beads (*K*_d_ = 10^−14^ mol/L) under very harsh conditions, including high concentrations of detergent, salt, or denaturing agents. Because of these major advantages over traditional methods, PL has been widely adopted to study PPIs, subcellular proteomes, cell type–specific proteomes, and protein–nucleotide interactions (29, 95, 120).

Another PL system, APEX or APEX2, engineered based on plant ascorbate peroxidases, has been mainly used in human cell lines and some dissected tissues (45, 63, 73, 96). The APEX system biotinylates proteins with the biotin-phenol substrate under oxidizing conditions (H_2_O_2_). This system prevails with its fast speed (the labeling can be done in 1 min) and its timely control upon H_2_O_2_ availability. In addition, the APEX system can also generate contrast in electron microscopy (73) and spatially map endogenous RNAs (32, 50). However, the APEX system has not been widely adopted in studies of plants due to low signal-to-noise ratio caused by the high background labeling from endogenous peroxidases. The poor membrane permeability of substrate biotin-phenol (which is much more hydrophobic than biotin) also restricts the use of APEX methods in complex tissues of plants (and animals). The stress response and potential toxicity induced by the substrate H_2_O_2_ further limit its use in plants. In this review, we focus on the biotin ligase–based PL system and its applications. A detailed review of the APEX system can be found in References 71 and 101.

In this review, we cover the most common or adaptable PL applications and their experimental designs. We then discuss different biotin ligases and enrichment choices. We also explore different quantification strategies involving mass spectrometric data analysis and offer an overview of the validation methods of the candidates discovered from PL-MS experiments. Lastly, we discuss the future prospects of PL-MS studies in plants.

## 2. PROXIMITY-LABELING MASS SPECTROMETRY APPLICATIONS TO STUDY PROTEIN–PROTEIN INTERACTIONS AND SUBCELLULAR/ORGANELLE AND CELL TYPE–SPECIFIC PROTEOMES

PL has emerged as a powerful technique, complementary to traditional methods (e.g., affinity purification and biochemical fractionation), to study protein interaction networks and to map subcellular and cell type–specific proteomes. In this section, we compare PL-MS to traditional methods, discuss the experimental designs, and demonstrate several application examples that can be potentially adapted by or directly applied to plant studies.

### 2.1. Using Proximity-Labeling Mass Spectrometry to Study Protein–Protein Interactions

PPIs underlie nearly every cellular process (106). Mapping PPIs in either protein assemblies or signaling pathways is essential for understanding the mechanisms of cellular processes and their roles in growth and development. While some of the interactions are stable, most of them are weak, transient, and restricted in a spatial and temporal manner. Accurate and comprehensive characterization of these dynamic interactions is critical in proteomic studies to annotate protein functions and to dissect the biological processes.

Affinity purification coupled with mass spectrometry (AP-MS) is most used in traditional methods for identifying PPIs. AP-MS has been used to map stable protein complexes successfully and, in some cases, to recover transient interactions using modified protocols (8, 42, 47, 81, 82). For instance, a quick and dirty procedure that includes quick binding and short and mild washes was performed to preserve the transient interactors (52). Alternatively, a low concentration of chemical cross-linkers was employed to detect the weak and transient interactions (111, 137). However, AP-MS experiments often result in the loss of weak and transient interactors during lysis and purification. Preserving protein interactions in AP-MS is even more challenging for less-soluble membrane-associated protein complexes or chromatin-associated protein complexes and for low-abundance protein complexes (39, 64).

PL-MS shows advantages over AP-MS in several aspects (39). Most prominently, the PL approach allows biotinylation labeling of both bait and prey in their native cellular environment such that the capture is independent of the affinity of the prey to the bait protein. Therefore, keeping the protein complexes in their native state during the affinity capture of biotinylated proteins is no longer necessary. Furthermore, compared to the affinity of antibody-mediated interactions used in most AP-MS such as the high-affinity tandem green fluorescent protein (GFP) nanobody (*K*_d_ = 3.6 × 10^−11^ mol/L) (34), a significantly higher affinity of streptavidin (*K*_d_ = 10^−14^ mol/L) allows the capture of biotinylated proteins under very harsh conditions (with high concentrations of detergent, salt, or denaturing agents) with a much greater efficiency. These conditions allow the near-complete extraction of the proteome, which significantly improves the input for binding. The high affinity also allows thorough washes to reduce contaminants (e.g., nonspecific binding proteins) and to remove detergents used in the initial steps of extraction and binding. Removal of detergents is particularly important for downstream on-bead digestion and liquid chromatography–coupled tandem mass spectrometry (LC-MS/MS) analysis as they are often contaminated with polymers that interfere with MS analysis.

A successful PL-MS experiment design for studying PPIs (Figure 1b) requires three critical parameters: (*a*) functional bait-fusion protein(s), (*b*) proper control(s), and (*c*) optimized labeling time. First, the bait protein is fused to a PL enzyme (Figure 1b) at either the N or C terminus and expressed under the bait protein’s native promoter at the endogenous level. A functional complementation analysis is a prerequisite to determine whether the bait-PL fusion protein is functional in its physiological state. Second, the choice of control is experiment dependent and can include (*a*) the PL enzyme alone or (*b*) the PL enzyme fused to an optional localization peptide under the same native promoter. Optional control constructs may also include the PL enzyme fused to an unrelated protein that localizes to the same compartment as the bait or to a truncated or mutated bait. It is important to ascertain whether the control fusion protein(s) and the bait protein have similar expression levels and share a similar subcellular localization pattern. For instance, if the bait localizes to the nucleus, then the control should also target the nucleus. The controls will generate a filter list of proteins, including endogenously biotinylated proteins, stochastically labeled proteins, and bead-bound nonspecific proteins (72, 138). Filtering out the stochastically labeled proteins that reside by chance in proximity of the bait from bona fide interacting partners is of particular importance, since the PL enzymes cannot distinguish between these two types of proteins. In addition, extra tags, such as the fluorescence, Myc, and Flag tags, are often included to examine localization and expression of both bait and control. Lastly, control over the biotin labeling time is equally critical for generating high-quality data. Depending on the abundance of the bait proteins, the duration of labeling needs to be experimentally determined to obtain optimal results. For example, a shorter time of biotin treatment (≤1 h) is preferred to get a snapshot of PPIs for more abundant proteins such as some kinases (58) and splicing factors (R. Shrestha & S.-L. Xu, unpublished observations), while a longer biotin treatment (up to several hours) may be needed for less-abundant bait proteins (e.g., transcription factors) (72). In summary, the above three parameters should be easily met in well-established plant models. For plant species in which transgenesis and mutants are not available, researchers should at least examine the proper localization of the bait-PL fusion proteins and express them close to the endogenous level to obtain bona fide interactors.

PL-MS has been adopted in plants and extensively applied in studying PPIs and mapping protein networks. For example, PL-MS has enabled the mapping of low-abundant protein transcription factor FAMA interactomes (72), the hard-to-enrich plasma membrane protein complex (3), and the nuclear envelope membrane protein complex (44, 116). PL-MS has also been superior for dissecting weak, transient interactions of signaling protein complexes, such as kinase protein/kinase substrates (58) or E3 ligase/substrates (139). These applications of PL-MS not only confirmed known interactors but also discovered many novel protein interactors, demonstrating the efficacy and potential of PL over traditional approaches in elucidating protein networks in plants.

### 2.2. Using Proximity-Labeling Mass Spectrometry to Study Subcellular Proteomes

Protein functions are closely correlated with their localization within the cell. Eukaryotic cells contain highly specialized membrane-bound and membraneless compartments that are critical to regulate various molecular complexes and biological processes in living organisms. Mapping the subcellular/organelle proteomes has long been a major interest in molecular biology to study their distinct and/or overlapping functions in both plants and animals (1, 21).

Various techniques, including organelle-specific biochemical fractionation and cell-wide biochemical fractionation (e.g., correlation profiling), have been attempted to study the proteome compositions of organelles and subcellular structures (21, 38). These methods take advantage of the differences in organelles’ density, size, or membrane properties to separate organelles via multiple centrifugations or detergent steps. Using these approaches, plant proteomics studies have been conducted on numerous membrane-bound compartments, including the chloroplast (7), mitochondria (100, 107), peroxisome (86), vacuole (105), and nucleus (91), along with other subcellular structures such as the plasmodesma (33) and nucleolus (87). Proteomics studies have also probed proteomes of different membranes, including the plasma membrane (130), Golgi membrane (85), and chloroplast envelope membrane (110). These attempts have provided invaluable insights into subcellular proteomics, organelle biogenesis, and functionality analysis. However, these traditional proteomics methods often suffer contamination from nontarget organelles, incomplete coverage/undersampling, and inconsistent assignment of protein localization to subcellular fractions. In addition, challenges remain in studying subcellular compartments and structures including membraneless organelles, which cannot be easily separated by traditional biochemical fractionations.

PL-MS has unique advantages in studying subcellular proteomics (29, 71). By targeting the PL enzyme to a subcellular location, the constituents within that compartment or subcellular structure are biotin labeled in a physiological context. The enrichment of these biotin-labeled resident proteins in PL-MS is straightforward and highly efficient using the high-affinity streptavidin beads, bypassing the requirement to preserve the integrity of organelles/subcellular structures during sample purification as in the conventional biochemical fractionation method.

Experiment designs (Figure 1c) for PL subcellular proteomics include fusing the PL enzyme to well-validated sorting motifs or localization peptides. For instance, the PL enzyme can be fused to a nuclear localization signal (NLS) to target the nucleus, to a nuclear export signal (NES) to target the cytosol, or to a transit peptide (e.g., from the Rubisco small subunit) to target chloroplast stroma (79). Alternatively, the biotin ligases can be fused to known resident proteins from different subcellular locations (40), albeit with the potential risk of interfering with function or localizing to microdomains (18). To ascertain whether most constituents within the compartment of interest are labeled, the bait proteins are often expressed constitutively in every cell. Proper controls typically include stand-alone biotin ligases or biotin ligases fused to a different localization signal or to resident proteins from other subcellular locations.

The PL-MS approach has been extensively applied in animals to study subcellular structures (39). Recently, large-scale subcellular mapping using BioID fused to 192 resident markers localized to over 20 intracellular compartments has been performed in human HEK293 cells (40). Go et al. (40) exploited multiple baits that localize to the same subcellular compartment to generate orthogonal data sets and assigned over 4,000 intercellular proteins.

Similar large-scale subcellular profiling has not been attempted in plants yet. Recently, Mair et al. (72) probed the nuclear proteome in *Arabidopsis* using PL-MS and identified proteins that were significantly enriched in nuclear assignment. To profile different compartments/subcellular structures, plant biologists can design PL experiments based on published subcellular proteomes that have been categorized by the Subcellular Localization Database for *Arabidopsis* Proteins (*SUBA*; https://suba.live) (41, 43, 117). More importantly, the highly active TurboID opens more possibilities for further investigations of subcellular proteomes in a spatial and temporal manner and other compartments that have been historically challenging to study, including membraneless, phase-separated, cytosolic, and nuclear granules and cell wall–embedded plasmodesmata. By virtue of TurboID, plant biologists can also extend current subcellular proteome studies to a wider range of plant species, including woody plants and crops, to dissect functions of organelles across plants at a systems level.

### 2.3. Using Proximity Labeling to Study Cell Type–Specific Proteomes

To study the unique biological functions and properties of individual cell types, in-depth protein expression analysis in different types of cells, particularly specialized cells (e.g., guard cells or bundle-sheath cells), is fundamental for investigating the unique functions of tissues and organisms (80).

Various types of tools have been developed to study the cell type–specific proteomes in both animals and plants. These tools include laser capture microdissection or fluorescence-activated cell sorting (FACS) of labeled cells in a tissue (9, 25, 26, 36, 48, 84) and less common methods such as mechanical separation and sequential protoplast generation (114). Together, these approaches have enabled cell type–specific proteomics studies in multiple tissues, including anther pollen (140), root (90, 144), leaf (114), and trichome (5).

Generating sufficient data sets from traditional methods, however, comes at a cost of time and effort to attain sufficient quality and quantity of isolated cells. As a noninvasive, time-efficient, and highly sensitive complementary tool, PL-MS has gained traction in studying cell type–specific proteomes (72, 112). By expressing the PL enzyme in a specific cell type, proteins therein can be tagged and efficiently isolated, allowing the proteomic workflow to be executed at the tissue level with minimized labor and time. More importantly, PL methods circumvent the challenges of obtaining enough pure cells and the automation workflow required in traditional methods.

To design PL-MS experiments for studying the proteome of specific cell type(s), the PL enzyme is expressed under a cell type–specific promoter and, in some cases, fused to a localization signal to target specific subcellular compartments. The control can be a PL enzyme expressed under either a different cell type–specific promoter or a constitutive promoter. For example, to study the guard cell–specific nuclear proteome, Mair et al. (72) expressed TurboID-NLS under the guard cell–specific FAMA promoter and used a constitutive promoter for the same fusion construct as the control. PL-MS not only identified known guard cell–associated transcription factors but also discovered many additional components that were identified by previous proteomics studies using 300 million guard cell protoplasts (141, 142).

PL-MS can be extended to many more cell types that are of high interest to plant biologists, including different root cells and the bundle-sheath cells in C_4_ plants (Figure 1d). Researchers can take advantage of cell type–specific promoters such as that of the nicotinamide adenine dinucleotide phosphate (NADP)-malic enzyme (bundle sheath–specific) and phosphoenolpyruvate (PEP) carboxylase (mesophyll-specific) (30, 128) to map the proteomes of these unique cell types and dissect their functions in the future.

## 3. MODULATED PROXIMITY-LABELING APPLICATIONS

Multiple modified PL approaches have been developed recently to tackle additional challenges and to increase the versatility of PL applications. In this section, we cover the split-PL system in studying conditional PPIs and hard-to-purify subcellular structures or compartments. We then discuss modified PL approaches that take advantage of CRISPR to study the protein–nucleotide interactome. Lastly, we will discuss a modular and versatile PL system controlled by a conditionally stable GFP-binding protein nanobody (GBP).

### 3.1. Split Proximity Labeling to Map Conditional Protein–Protein Interactions and Localized Proteome

**Figure 2:**
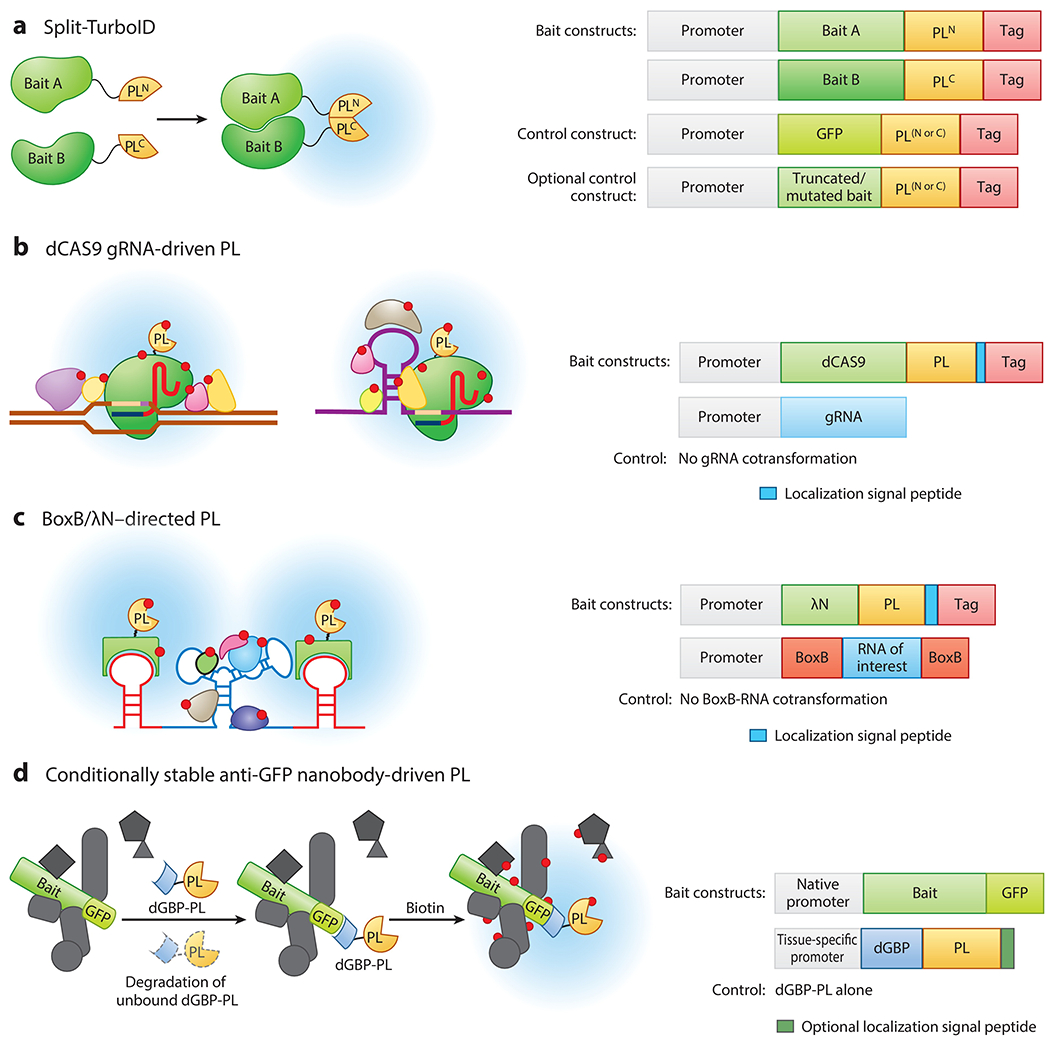
Modulated PL applications. (*a*) Schematic of the split-PL approach and suggested design of the constructs. The PL enzyme is divided into two inactive fragments (PL^N^ and PL^C^) that fail to bind unless brought together by either protein–protein interactions or organelle–organelle interactions to reconstitute biotinylation activity. Choice of promoters is experiment dependent (e.g., native promoters are preferred for conditional protein–protein interactions). (*b*) Diagram depicting CRISPR-driven PL for protein-DNA or protein–RNA interaction studies. The PL enzyme fused to the nuclease-deficient dCas9 protein (or dCas13 or dCasRx) is targeted to a specific genomic locus or RNA of interest via a gRNA and biotinylates proteins in its vicinity. (*c*) Scheme showing BoxB/*λ*N-directed PL to detect the RNA-binding protein. Engineered RNA is composed of BoxB stem loops flanking the RNA motif of interest. The PL enzyme is fused to the *λ*N peptide and targeted to the engineered RNA via the *λ*N-mediated binding to the BoxB stem loop. Upon biotin treatment, the PL enzyme will biotinylate the RNA-binding proteins in its vicinity. Two BoxB stem loops are included in the engineered RNA to increase the sensitivity of biotinylation labeling. (*d*) Illustration of the conditionally stable dGBP-PL system. The dGBP-PL fusion protein transgenic lines can be crossed with existing transgenic GFP-tagged lines for PL studies. Proximity biotinylation is initiated when dGBP-PL is stabilized upon binding to the bait-GFP fusion protein in cells containing both transgenes. By contrast, the unbound dGBP-PL is unstable due to its rapid degradation by the ubiquitin proteasome system; thus, the background labeling is minimal. The dGBP-PL construct can contain an optional localization signal peptide and be expressed in the same tissue as the bait to obtain optimal results. Biotin is represented by red circles. The blue region denotes the biotinylation radius. Abbreviations: CRISPR, clustered regularly interspaced short palindromic repeats; dGBP-PL, anti-GFP nanobody–driven PL; GBP, GFP-binding protein nanobody; GFP, green fluorescent protein; gRNA, guide RNA; PL, proximity labeling.

Consisting of two inactive fragments of the biotin ligase (BioID or TurboID) that can be reconstituted through PPIs or organelle–organelle interactions, split-PL has enabled the study of conditional interactomes and proteomes of subcellular structures that are difficult to access by conventional PL approaches (17, 27, 61, 104, 115) (Figure 2a).

In cells, an increasing number of proteins have been identified as moonlighting proteins that associate with multiple complexes. Deconvoluting their interactomes and assigning them to specific complexes will be key to dissecting their distinct and/or overlapping functions (46). Split-BioID has been applied to assign specific complexes to moonlighting proteins. For instance, Argonaute 2 (Ago2) is involved in microRNA (miRNA)-induced silencing and known to be part of at least two functionally distinct complexes, including the miRNA-induced silencing complex (miRISC) and the RISC-loading complex (RLC). Schopp et al. (104) generated constructs containing Ago2 paired with two different known binding partners, Dicer and TNRC6C from the miRISC and RLC, respectively (NBirA*-Dicer/CBirA*-Ago2 and NBirA*-TNRC6C/CBirA*-Ago2), and used NBirA*-GFP as the control. Split-BioID probed the proteomes of distinct functional complexes containing Ago2 and identified novel candidates. Similarly, split-BioID was employed to map interactomes for a specific heterodimeric protein phosphatase complex (27).

Split-PL has also been utilized to map some challenging localized proteomes (17, 61, 115) with greater specificity in the targeting of biotinylated locations. Some localized proteomes (e.g., endoplasmic reticulum–mitochondrial contact sites) are very hard to map selectively because available characterized resident proteins also localize to other regions, resulting in nonspecific and convoluted mapping when fused to full-length PL. Meanwhile, traditional methods such as sequential centrifugation often suffer from contamination from other organelles and lack reproducibility. By combining split-PL with the chemically inducible FRB-FKBP dimerization system, this contact site has been mapped with a higher specificity (17, 61). Similarly, split-PL has been adopted to study hard-to-reach astrocyte–neuron junctions (115).

In the future, Split-PL can be easily adapted to the plant system to study conditional PPIs and organelles that are yet to be mapped, including the photobody (126) and plasmodesmata (65). In addition, split-PL can be combined with an on/off switch to control targeted proteome mapping more tightly. For instance, split-PL can be combined with a light switch to probe the photobody compositions using the phytochrome–phytochrome-interacting factor (PIF) protein systems, given that phytochrome and PIF proteins move to the photobody upon their light-dependent interaction. A caveat of the split-PL system, however, is its lower kinetics than those of the full-length PL. For instance, Cho et al. (17) showed that split-TurboID required 4 h of biotin treatment to obtain comparable results with those of TurboID using 1 min of biotin treatment.

### 3.2. CRISPR-Driven Proximity-Labeling Mass Spectrometry to Study the Protein–Nucleotide Interactome

Coupling CRISPR technology (143) with PL-MS has recently emerged as a valuable complementary approach to comprehensively map local chromatin interactions and architecture in situ (37, 78, 103). Gene expression is controlled at specific regions of genomic DNA by the concerted action of myriad transcriptional regulatory proteins, which either activate or repress the transcription of target genes. Tremendous effort has been made to map these transcriptional regulatory complexes on a particular chromatin locus of interest to study local chromatin composition and architecture. Chromatin immunoprecipitation (ChIP) (35), a conventional approach, has provided information on both single-locus and genome-wide distribution of transcription factors but is often limited to examining a single previously defined transcription factor in each experiment. Chemical cross-linking and precipitation with complementary DNA probes have been employed to directly analyze chromatin complexes but often suffer from the loss of cellular and/or chromatin context (28, 53).

PL-MS shows great potential in mapping protein–DNA interactomes. Guided by single-guide RNAs (sgRNAs), a nuclease-deficient dCas9-fused biotin ligase targets a specific genomic locus (Figure 2b) and biotinylates proteins in close proximity to the target locus (103). To ensure the specificity of dCas9-PL, ChIP–quantitative real-time polymerase chain reaction (qPCR) is a necessary quality-control step to examine the target locus enrichment by pulling down either dCas9-PL or biotinylated proteins followed by qPCR. The control experiment contains no sgRNA co-transformation for generating a filter-out list consisting of off-target chromatin interactors and other usual contaminants (e.g., nonspecific binding proteins and endogenous biotinylated proteins). Similar approaches using APEX enzymes also successfully mapped the subnuclear proteomic landscapes to predefined genomic loci (37, 78).

The CRISPR-driven PL approach can be easily adapted to plant systems to study chromatin interactors. Several reporter lines, such as the stably transgenic DR5 and TCSn reporter lines used for auxin and cytokinin responses, respectively (125, 145), can be utilized to test their efficacy; these reporters contain tandem regulatory elements that can boost the detection sensitivity. CRISPR-PL and specific gRNAs can be designed to probe the chromatin interactors at these loci, and we would expect to detect AUXIN RESPONSE TRANSCRIPTION FACTORS (ARFs) or type B *Arabidopsis* response regulators (ARRs). This CRISPR-driven PL-MS approach can be used to study the regulatory components of multiple plant genes of interest that are subjected to tight controls in response to different biotic or abiotic responses. However, whether all the identified chromatin interactors from PL-MS are specific to the genomic elements remains unknown but can be determined in the future with the use of large collections of data.

Using the same principle, CRISPR-coupled PL-MS has also been adapted to map the protein–RNA interactomes (Figure 2b). Yi et al. (133) developed the CRISPR-assisted RNA–protein interaction detection (CARPID) method to probe binding proteins of specific long noncoding RNA (lncRNA) X-inactive-specific transcript (XIST) in a native cellular context. To examine the targeting specificity guided by sgRNA, a functional CasRx fused to biotin ligase was utilized to confirm the cleavage in target RNA. This CARPID approach identified not only known validated XIST interactors but also many more novel interacting proteins (133).

Alternatively, to probe RNA-interacting proteins, some researchers have utilized BoxB-λN or MS2-MCP systems to target PL enzymes to an engineered RNA of interest fused with a BoxB stem loop (or MS2 stem loop) to induce biotinylation of RNA-binding proteins (77, 92, 135) (Figure 2c). Such methods map proteins that bind to engineered RNA but not those that bind to endogenous RNA but may have advantages for studying RNA-binding proteins in more limited regions of RNA.

### 3.3. A Modular System Using a Conditionally Stable GBP–Proximity Labeling Method to Map Protein–Protein Interactions

To enhance the versatility of the PL system, a modular system has been developed that can take advantage of existing transgenic GFP lines instead of generating transgenic lines containing each bait-PL fusion (and control) for each protein of interest. To screen for Cavin-associated networks, the Hall group (129) combined TurboID with a conditionally stable GFP-binding protein [destabilized GBP (dGBP), a 14-KDa nanobody] to create a destructive GFP-directed PL line in zebrafish and outcrossed this line to existing transgenic lines that contain native promoter-driven Cavin bait proteins fused with a GFP variant. Notably, the dGBP-PL fusion protein is nearly undetectable due to its rapid degradation via the ubiquitin proteosome system, thereby generating minimal background labeling within target cells in vivo. Biotinylation only starts when dGBP-PL stabilizes upon binding to the bait-GFP fusion in cells expressing both bait-GFP and dGBP-PL (129) (Figure 2d). This system enhances the versality of PL applications by allowing the proteomic mapping between different bait proteins with available GFP fusion transgenic lines.

It is tempting to adapt this system to plants for proteomic mapping between different proteins, given that many transgenic GFP lines are available within the plant community. Whether the conditionally stable nanobody, when not bound by the antigen, is also rapidly degraded by the ubiquitin proteasome system in plants remains to be tested. We predict, however, that it will work in a similar manner since multiple nanobodies have been tested in the plant system (127). To achieve optimal results, multiple transgenic lines containing dGBP-TurboID (or other biotin ligases) fused with different localization signals should be created and crossed with existing GFP-tagged lines that have similar subcellular localizations. The promoter used for dGBP-TurboID may also be optimized to achieve desirable results.

## 4. CHOICES OF BIOTIN LIGASES

**Figure 3:**
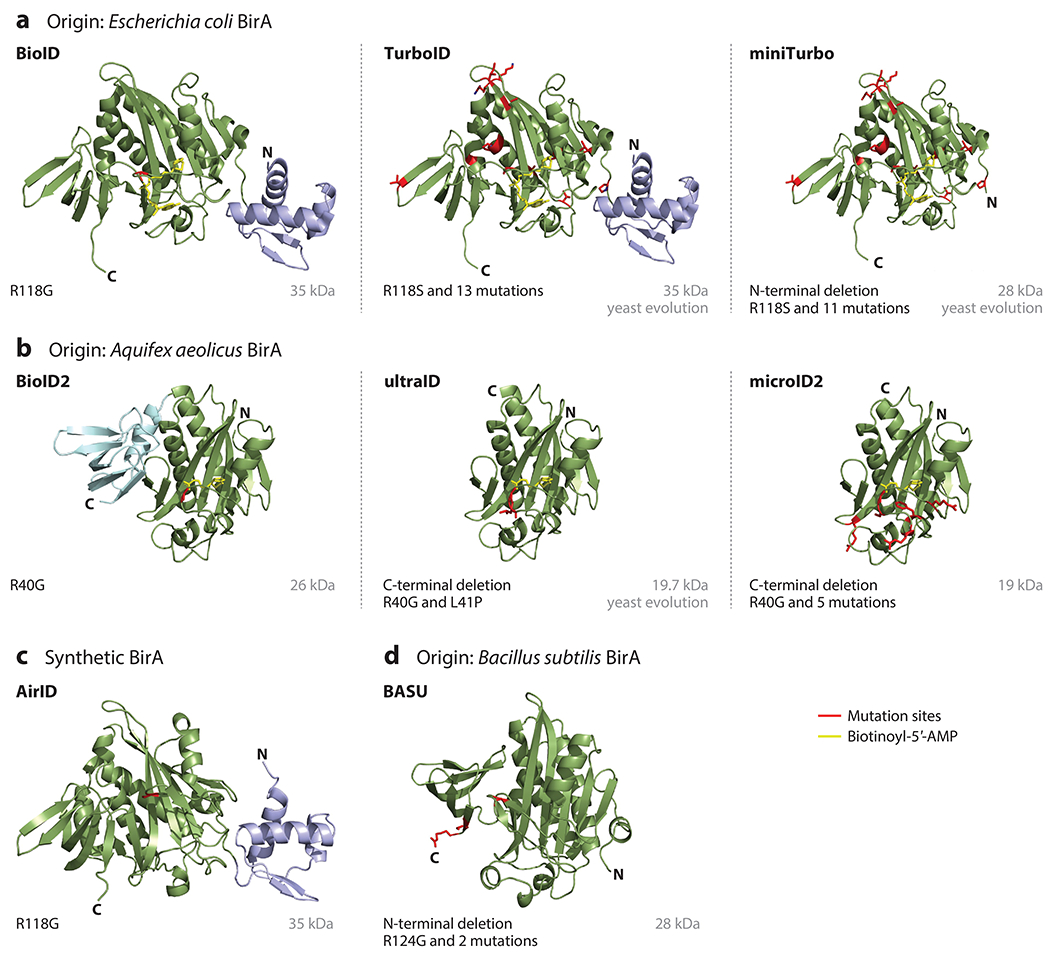
Choices of engineered biotin ligases. (*a*) Structure of biotin ligases engineered from *Escherichia coli* BirA, including BioID (BirA*) with an R118G mutation, TurboID with R118S and 13 additional mutations, and miniTurbo with R118S and 11 additional mutations and a truncated N terminus. (*b*) Structure of biotin ligase variants containing an R40G mutation engineered from *Aquifex aeolicus* BirA, including BioID2, ultraID with a truncated C terminus and an additional L41P mutation, and microID2 with both a C-terminal deletion and 5 additional mutations. This family differs from *E. coli* BirA due to the absence of the N-terminal domain. (*c*) Structure of AirID, a synthetic version of BirA with R118G mutation using ancestral enzyme reconstruction and site-directed mutagenesis. (d) Structure of BASU engineered from *Bacillus subtilis* BirA with R124G and 2 additional mutations as well as a truncated N terminus. Mutation sites are highlighted in red, and the biotinoyl-5′-AMP is depicted in yellow. Structures in panels *a* and *b* were generated based on Protein Data Base structures 2EWN and 3EFS, respectively; structures in panels *c* and *d* were generated by AlphaFold; visualization of structures was done in Pymol.

We group the major available engineered biotin ligases based on their origins (Figure 3): (*a*) BioID, TurboID, and miniTurbo, derived from *Escherichia coli* BirA (10, 55, 99) (Figure 3a); (*b*) BioID2, ultraID, and microID2, derived from *Aquifex aeolicus* BirA (10, 49, 60) (Figure 3b); (*c*) AirID, a synthetic BirA* designed de novo using ancestral enzyme reconstruction and site-directed mutagenesis (54) (Figure 3c); and (*d*) BASU, derived from *Bacillus subtilis* (92) (Figure 3d). Mutation at the conserved residue arginine (R) in the catalytic domain is shared among all versions of all the engineered biotin ligases and is most critical as it engenders a 100-fold greater dissociation constant for biotin and an approximately 400-fold higher dissociation rate for the reactive biotin intermediate (biotinoyl-5′-AMP) compared to wild-type BirA (62). When biotinoyl-5′-AMP is no longer retained, the biotin ligases become promiscuous and label proteins in the vicinity (19). Supplemental Table 1 summarizes the labeling time and working temperatures for each biotin ligase, tested working organelles in both animals and plants, and tested plant species. Significant engineering effort has been devoted to improving the catalytic efficiency and activity of the biotin ligases, reducing their size, and minimizing the background labeling.

As the first developed biotin ligase with promiscuous protein-labeling activity, BioID (BirA*) has been extensively applied in studies in a variety of subcellular organelles and in protein network mapping (13, 20, 40) (Supplemental Table 1). However, with an optimum working temperature at 37°C, BioID has a slow labeling kinetics with 18–24 h or more labeling durations, which affects its temporal resolution, as BioID labels all the proteins in proximity over time rather than giving a snapshot of protein interactors. BioID has a limited applicability in model organisms, for example, flies, worms, and plants (10, 72).

TurboID, engineered via a directed evolution of BioID by the Ting group (72), has the highest activity of all biotin ligases and the broadest working temperature for model species (3, 10). With its rapid labeling kinetics in a time scale of minutes, TurboID is suitable for studying spatial and temporal cellular dynamics and subcellular or organelle proteomes (3, 72, 94, 113). The drawback of TurboID, however, is its high background labeling from endogenous biotin due to its higher sensitivity to biotin. To address this issue for applications that specifically need lower background labeling, the Ting group (72) also engineered miniTurbo, which allows for more control over background labeling. Hence, miniTurbo would potentially be beneficial in studying plant tissues that have higher endogenous biotin levels than average (e.g., mature green siliques) (108). The overall activity of miniTurbo, however, is lower than that of TurboID and is less stable in some experimental systems (10, 129).

The second group includes BioID2, ultraID, and microID2 (Figure 3b), all of which were engineered from a biotin ligase of the thermophilic bacterium *A. aeolicus* (49, 57, 60). This family differs from *E. coli* BirA due to the absence of the N-terminal domain (Figure 3b). BioID2 has been utilized in mapping protein networks and subcellular proteomics but suffers from slow labeling kinetics (4, 11, 16, 57, 69, 134). Recently, a C-terminal truncation of BioID2 combined with directed evolution was used to create ultraID (60). Similarly, a C-terminal truncation of BioID combined with site-directed mutagenesis was used to create microID2 (49). Both ultraID and microID have higher activity levels than BioID2. Particularly, the smaller size of ultraID (19.7 kDa) and microID2 (19 kDa) may be advantageous to reduce the potential interference at the fusion protein for its proper folding, function, and localization. Notably, microID2 has been shown to be unstable in transgenic lines (49). Compared to other biotin ligases, this group shows optimum activity and thermal stability at higher temperatures, which may be suitable for applications in species such as desert plants or thermophilic algae that grow in high temperatures.

AirID (Figure 3c) is a new biotin ligase enzyme developed by the Sawasaki group (54) by using ancestral enzyme reconstruction and site-directed mutagenesis. AirID shows 82% sequence similarity to BioID but has a higher biotinylation activity. However, with a requirement of 3–6 h of labeling, AirID still exhibits lower enzyme kinetics than TurboID. The activity of AirID has been tested in mammalian cell cultures (131) but not in plants yet. BASU (Figure 3d) was developed by the Khavari group (92) from *B. subtilis* BirA by introducing a truncation of the N terminus and three mutations, including one R124G mutation, to its reactive biotinoyl-5′-AMP binding motif. BASU has a similar enzyme activity as BioID and BioID2 (10); its activity has been tested in mammalian cells (10) and in zebrafish (129) but not yet in plants.

Researchers need to determine which biotin ligases work best for their species as well as their specific experiments. TurboID and miniTurbo have been tested to work effectively in multiple plant species, including *Arabidopsis*, tobacco, tomato, liverwort, maize, and citrus (Supplemental Table 1). Whether TurboID and other biotin ligases are suitable for use in more plant species and organelles remains to be tested. Surprisingly, both TurboID and miniTurbo fail to work in the cytosol or nucleus of green algae, but a less-active intermediate G3 version (prior to TurboID) derived from yeast-directed evolution (10) works (Grossman lab and Onishi lab, unpublished observations); the exact reason for the failure of TurboID/miniTurbo in this context remains to be determined. Thus, it will be of interest to examine if other biotin ligases such as AirID, microID2, or ultraID work better than the G3 version in green algae. Similarly, for plant species in which TurboID has not been tested, researchers may initiate pilot experiments to compare different biotin ligase choices and determine which one works best. However, whether biotin ligases work in more organelles remains to be tested. For instance, TurboID has been extensively tested in several organelles and subcellular structures, but whether TurboID and other biotin ligases work in a vacuole with low pH or in the extracellular domain with low ATP concentration remains undetermined.

TurboID has the highest activity, so it is often the top choice for mapping proteomes in a temporal manner. Yet it may not always be the best choice since it has a higher propensity for using endogenous biotin than other biotin ligases. For instance, the high self-biotinylation of the bait-TurboID fusion protein may potentially inactivate the bait protein and/or interfere with the bait’s function if surface-exposed biotin-modified lysine sites are essential to functionality. Additionally, if the turnover rate for some prey proteins is very low, then a relatively high background labeling introduced by TurboID may be problematic for temporal mapping, as the labeling may already be saturated before biotin and/or stimulus treatment. Note that biotin is a critical catalytic factor for several key carboxylases in plants (15); reducing the endogenous biotin level without causing stress to the plants can therefore be a daunting task. Thus, other biotin ligases, despite being less active than TurboID, may in some cases be a better choice with lower background labeling or less toxicity.

## 5. EFFECTIVE ENRICHMENT STRATEGIES FOR DETECTING BIOTINYLATED PROTEINS

Effective enrichment of biotinylated proteins is key for the success of PL experiments. The enrichment can be done at the protein or peptide level. Overall, the protein-level enrichment prevails with its simplicity; the peptide-level enrichment excels at the reliability of its data. Here, we describe the workflows of the two strategies and compare their respective strengths and weaknesses (Table 1).

### 5.1. Enrichment of Proximity-Labeled Proteins at the Protein Level

**Figure 4:**
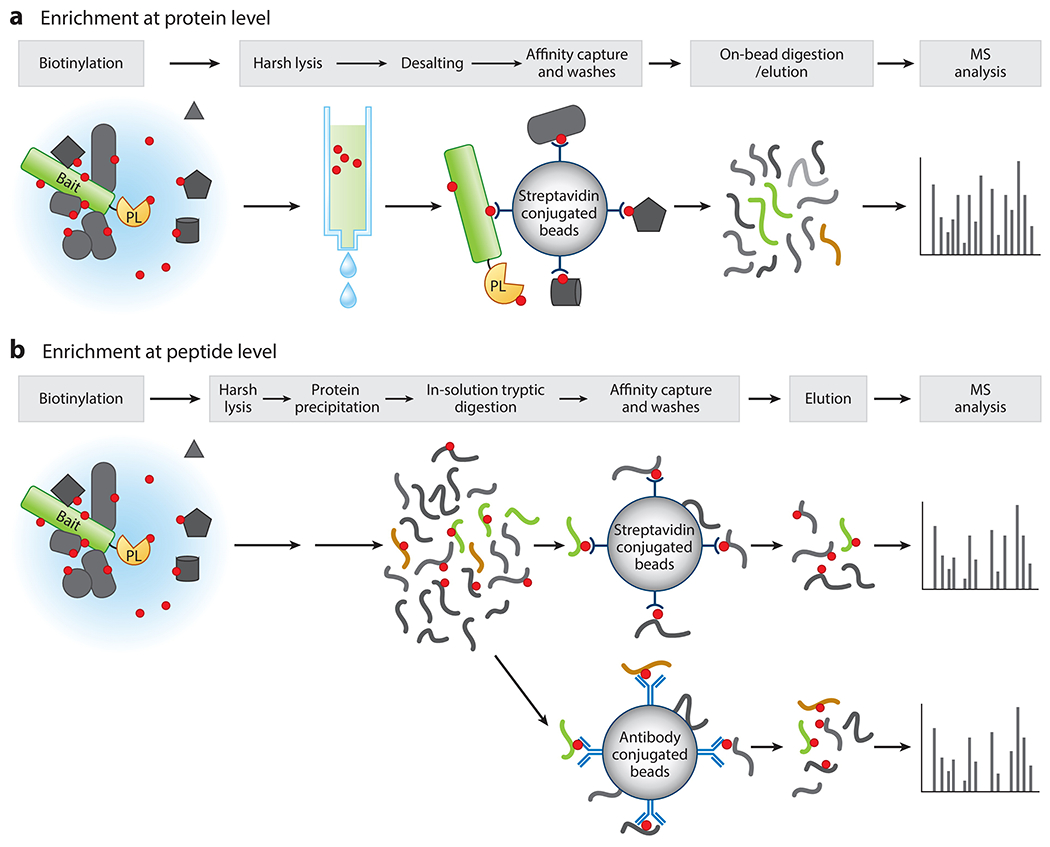
Comparison of protein- and peptide-level enrichment for the capture and detection of proximity-labeled biotinylated proteins. (*a*) Depiction of the workflow of protein-level enrichment of biotinylated proteins in plant systems. Biotinylated samples are subjected to harsh lysis and the removal of free biotin by a size-exclusion column, followed by the affinity capture of biotinylated proteins using streptavidin beads and elution by on-bead tryptic digestion. The eluate is further digested by trypsin prior to LC-MS/MS analysis. (*b*) For peptide-level enrichment of biotinylated proteins, biotinylated samples are extracted after harsh lysis, followed by protein precipitation. The proteins are then subjected to in-solution tryptic digestion to peptides. Biotinylated peptides are subsequently captured by either streptavidin beads followed by harsh elution or antibody-coated beads followed by mild elution prior to LC-MS/MS analysis. Biotin is represented by red circles. The blue region denotes the biotinylation radius. Abbreviations: LC-MS/MS, liquid chromatography–coupled mass spectrometry; MS, mass spectrometry; PL, proximity labeling.

Most of the published PL experiments have adopted enrichment at the protein level, in which streptavidin beads capture intact proximity-labeled proteins in the binding step (67, 72) (Figure 4a). Because the streptavidin has an extraordinarily high binding affinity to biotin (*K*_d_ = ~10^−14^ mol/L), the lysis, binding, and washes can be done in very harsh conditions (with detergent, salt, or denaturing reagents). However, the high affinity of streptavidin to biotin also poses a challenge to elution. To procure an efficient elution and to enable highly biotinylated proteins to be eluted at the same rate as less-biotinylated proteins, most labs perform on-bead digestions for elution in which the biotinylated proteins are eluted from the beads using a short (~3-h) trypsin digestion, followed by overnight full digestion and analysis via LC-MS/MS (Figure 4a). Overall, the protein level enrichment is simple and straightforward. The drawback to this approach, however, is that the biotinylated peptides are rarely detected, mainly because the samples are overwhelmed by nonbiotinylated peptides and the trypsin digestion elution is too mild to break the biotinylated peptides’ binding to the high-affinity streptavidin beads. To recover the remaining biotinylated peptides on the beads, Arora et al. (3) attempted a second-step elution using biotin and boiling with strong acid and were able to detect some biotinylated peptides from a small proportion of enriched proteins.

When using the protein-level enrichment strategy in plants, avoiding high concentration biotin treatment is important because free biotin competes with binding to streptavidin beads, leading to a significantly reduced capture (3, 58, 72). Meanwhile, conventional washes to remove free biotin used for human cell lines (22) are often not sufficient for plants. Hence, a desalting step using size-exclusion columns such as PD-10 to remove the free biotin and less than 50 μM of biotin treatment are strongly recommended for experiments (3, 72). Additionally, removing free biotin through protein precipitation before enrichment has also been reported (83) but has remained challenging for the downstream streptavidin capture due to incomplete or inconsistent resolubilization of precipitated proteins.

### 5.2. Enrichment of Proximity-Labeled Proteins at the Peptide Level

Several labs, including ours, have attempted to enrich proximity-labeled proteins at the peptide level in a process in which streptavidin beads capture biotinylated peptides (instead of biotinylated proteins) in the binding step (Figure 4b). Compared to protein-level enrichment, this approach has several advantages. First, detection of biotinylation sites provides direct and highly confident evidence of PL (56, 123). Second, it allows for a higher concentration of biotin treatment as biotin can be removed during the protein precipitation step. Third, in some cases such as for the subcellular proteome, the biotinylation site information offers insights on the topology of proteins. The peptide-level enrichment approach uses conditions similar to those mentioned above to lyse the tissues, but the extracted proteins are first precipitated and subjected to trypsin digestion. The resulting peptides are then used as input for streptavidin bead capture (102) or antibody enrichment (56, 123), followed by LC-MS/MS analysis. Alternatively, biotinylated peptides are enriched using an engineered avidin-like protein that has reversible biotin-binding capacity (76).

Peptide-level enrichment is considered to be less sensitive than protein-level enrichment due to its much lower abundance compared to nonbiotinylated peptides (56, 123). In MS, identifying any peptide from a protein is sufficient to determine the protein accession number. However, our lab found that the two enrichment strategies produce nearly comparable results after optimizing the peptide enrichment protocols (>50% of peptides were identified as biotinylated peptides). We noticed that the overall complexity of the samples is dramatically reduced after peptide-level enrichment, which in turn increases the chance for detection of biotinylated peptides (R. Shrestha & S.-L. Xu, unpublished observations).

## 6. QUANTIFICATION STRATEGIES USED IN PROXIMITY LABELING–MASS SPECTROMETRY

**Figure 5:**
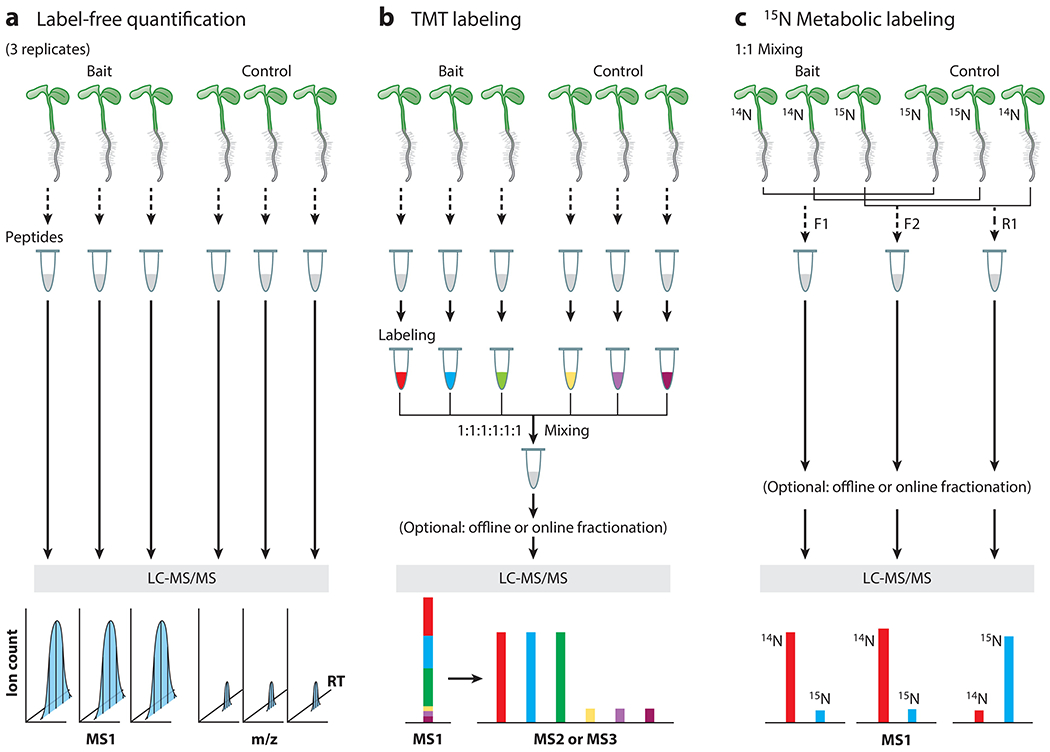
Schematic overview of the experimental workflow of three major quantification approaches in PL-MS experiments. Three biological replicates are required in each approach. (*a*) For label-free quantification, peptides from PL samples are directly measured by MS analysis. Protein abundance is quantified using the sum of XICs of peptides at the MS1 level. (*b*) For TMT isobaric labeling multiplexing quantification, each PL sample is labeled at the peptide level with the unique isobaric tag of the reagent, and the resulting barcoded samples are mixed in equal parts for simultaneous MS measurement. Quantification is based on the intensities of the yielded reporter ions at either the MS2 or MS3 levels. (*c*) For ^15^N metabolic labeling, plants are grown in ^14^N and ^15^N media and mixed to generate two forward samples, F1 and F2 (^14^N-bait/^15^N-control), and one reverse sample, R1 (^15^N-bait/^14^N-control), at the beginning of sample processing. Quantification is done by comparing the intensities of ^14^N and ^15^N peaks using XICs at the MS1 level. In panels *b* and *c*, either offline or online fractionations can be incorporated to increase proteome coverage prior to MS analysis. Abbreviations: LC-MS/MS, liquid chromatography-coupled mass spectrometry; MS, mass spectrometry; m/z, mass to charge; PL, proximity labeling; RT, retention time; TMT, tandem mass tag; XIC, extracted ion chromatogram.

Protein quantification is an integral step in the PL-MS workflow to identify enriched targets in PL-MS samples. The specific choice of quantification strategy depends on the experimental aims and designs, the studied species, and the cost. Here, we review the three most used quantification methods, including label-free quantification (LFQ), multiplexed tandem mass tag (TMT) isobaric labeling, and ^15^N metabolic labeling (Figure 5). We compare the strengths and weaknesses of each method in Table 2. We also briefly discuss the normalization method used in PL-MS analysis to achieve accurate and high-throughput quantification. To achieve better results, all quantification data should be acquired on high-resolution/high-accuracy instruments.

### 6.1. Label-Free Quantification

LFQ is the method most used in PL-MS quantification (44, 72, 138) due to its simplicity, low cost, and unlimited numbers of samples being compared (23). In addition, the downstream data processing following data acquisition is straightforward, using the publicly available software Maxquant/Perseus for data search and statistical analysis, and can be performed by most users with minimum training (24, 121, 122).

The LFQ method does not restrict the type of plant species, growth conditions, and growth duration that may be used. Typically, after trypsin digestion, each PL sample is directly injected for MS analysis (Figure 5a). The LFQs depend on the accurate determination of extracted ion chromatograms (XICs) of peptides at the MS1 (precursor ion) level between the runs. Peptide ion signals from each protein are summed to increase the sensitivity of quantification and are compared between baits and controls to determine enrichment. A good LFQ relies heavily on a high-standard MS instrumentation condition, demanding similar sensitivity in mass spectrometers and minimum shifts of retention time in liquid chromatography across all data acquisitions within the same experiment. LFQ generally has a higher amount of variance than TMTs or ^15^N metabolic labeling and does not allow fractionations for complex samples due to quantification complications (31, 93).

### 6.2. Multiplexed Tandem Mass Tag Isobaric Labeling Quantification

Isobaric tagging reagents TMTs (31, 93) have been employed for relative quantification of a large number of samples used in PL-MS experiments (17, 138). For concurrent MS analysis of multiple samples, each PL sample is barcoded by a unique isobaric tag of the reagent at the peptide level, and the resulting labeled samples are mixed for MS analysis (Figure 5b). Up to 18 samples can be simultaneously analyzed using the most recent TMT18pro kits (68). Within a TMT labeling set, all mass tagging reagents (barcodes) have an identical nominal mass and chemical structure composed of a mass reporter, a spacer arm, and an amine-reactive *N*-hydroxysuccinimide (NHS) ester group. This NHS ester group conjugates and tags the peptide via a covalent reaction to the peptide’s N terminus and lysine residue(s). Since different tags only vary in terms of distribution of the heavy isotopes (^13^C and ^15^N) within the reporter ions and mass normalization group, the same peptide from different samples will possess the same precursor mass after labeling and coelute in chromatography, appearing as a single peak in the MS1 (precursor ion) scan. After MS/MS (MS2) fragmentation of the precursor, each TMT will generate a unique reporter mass (e.g., 126 to 131 Da from TMTsixplex kits) in the low-mass region of the high-resolution MS/MS spectrum (Figure 5b). The abundance of these reporter ions is used for the relative quantification of peptide/protein abundance from each sample. Alternatively, the reporter ions can be generated and reported at the MS/MS/MS (MS3) level (in which multiple MS2 fragment ions are co-isolated and cofragmented to generate the MS3 spectrum). Quantifying at the MS2 level provides more sensitivity, while quantifying at the MS3 level provides more accuracy (118).

Like LFQ, TMT isotope labeling quantification can be applied to different species without restrictions on growth conditions or duration. Because samples are mixed and measured together, TMT labeling provides more accurate quantification than LFQ and enables a higher coverage (112) because it allows extensive fractionations, such as strong cation exchange or high pH reverse-phase high-performance liquid chromatography.

### 6.3. ^15^N Metabolic Labeling

^15^N metabolic labeling quantification is another method for PL-MS quantification in plants (58). In this workflow (Figure 5c), ^15^N-containing salts are incorporated into the plant proteins in vivo, contributing to a difference in mass measured at the MS1 (precursor) level for relative protein quantification (109). Samples are typically reciprocally labeled in replicates to reduce systematic error. ^14^N and ^15^N pairs are quantified at the MS1 (precursor) level using extraction ion chromatography, and then the median ratio of all quantified peptide pairs from each protein is reported (109). As the samples can be mixed at the beginning of sample processing, the ^15^N metabolic labeling significantly reduces preparative and analytical variabilities, thereby enabling more accurate quantifications compared to both LFQ and TMTs. Like TMTs, ^15^N metabolic labeling allows for extensive fractionation of the complex samples to achieve a high coverage. The drawback of the ^15^N metabolic labeling, however, is its restrictions on the type of plant species and the duration of plant growth (109).

### 6.4. Data Normalization

Normalization is a critical step in all quantifications. In most of the PL experiments in which a large collection of background proteins is detected, global normalization is sufficient to generate accurate quantification as it assumes the majority of all the detected proteins are similar across samples. However, for sample types that have overall much higher and more distinct signals than others, local normalization has to be applied to achieve better quantification. For instance, the majority of proteins identified from a ubiquitously expressed TurboID experiment are differentially abundant from those identified from a guard cell–specific TurboID (72), and a global normalization results in an overall reduction of signals and biased normalization and must therefore be corrected using a local normalization method such as LOESS-R (14).

## 7. VALIDATION OF CANDIDATES FROM PROXIMITY LABELING–MASS SPECTROMETRY EXPERIMENTS

PL-MS experiments, like most other discovery proteomics studies, generate a wealth of data. Identifying the known interactors or well-established markers often assures the quality of the data, and validating novel candidates is a necessary and exciting step to prove their biological relevance. Genetic perturbations (e.g., knockdowns/knockouts and overexpression) of candidate proteins from PL-MS experiments are common approaches to validate their biological functions. These genetic manipulations are expected to cause a phenotype that is similar to or opposite of that of the bait protein from PPI mapping (139), alter the organelle functions from subcellular proteomes (17, 124), or induce chromosome activation or inactivation from protein–DNA interaction mapping (133). To validate direct PPIs, bimolecular fluorescence complementation (BiFC) and yeast two-hybrid assays are commonly used (72, 139). Alternatively, in AP-MS or reciprocal PL-MS, switching the identified candidate to bait can also be utilized to validate the interactions. Less common approaches such as proximity ligation assay (6) can also be used to confirm PPIs. In addition, fluorescence imaging of newly identified candidates to verify their localization is often recommended for validation in subcellular and cell type–specific proteome studies. For candidates discovered from protein–DNA/RNA interactome PL-MS experiments, orthogonal approaches such as fluorescence in situ hybridization (e.g., immunoFISH), cross-linking immunoprecipitation sequencing (CLIP-seq), and RNA immunoprecipitation (RIP)-qPCR will serve the purpose for validation (133).

## 8. FUTURE PROSPECTS

As the development of PL-MS gains traction in plant research, mapping more dynamic interactomes and subcellular proteomes, particularly those that occur in response to environmental cues, will help address more biological questions. For instance, what is the protein interface for driving the light intensity–dependent movement of chloroplasts inside of cells (51) or for providing the precise guidance of pollen tube growth by the ovule during pollination (12)? We envision that elegant PL-MS experiments can be designed to answer these questions.

Coupling the power of PL-MS with posttranslational modification (PTM) studies will help to elucidate the regulatory mechanisms for dynamic changes (29). PTMs such as phosphorylation and *O*-GlcNAcylation have been shown to affect protein localization, PPIs, and protein–DNA interactions (132). Correlating and linking the PTM changes with protein changes discovered from PL-MS studies will be important. Recently, PL-MS has been coupled with a PTM reader to allow PTM-driven labeling in subcellular space and to monitor the dynamic changes of protein complexes. For example, a GlycoID-coupling miniTurboID with an *O*-GlcNAc reader (GafD, an *O*-GlcNAc binding protein) was created to track the dynamic changes of protein complexes associated with *O*-GlcNAc-modified targets (70).

Coupling PL-MS with cross-linking mass spectrometry (XL-MS) will also be a promising approach to reveal the architecture of protein complexes. While PL-MS provides a parts list for interactomes and subcellular proteomes, XL-MS offers connectivity and structures of the protein complexes (136). In XL-MS, after interacting proteins react with bifunctional cross-linking reagents to become cross-linked, proteins are extracted and subsequently digested to peptides and subjected to MS analysis. The identification of the cross-linked peptides will not only present direct evidence of connectivity between proteins but also map interacting domains as well as the structure of protein complexes. These PL-MS and XL-MS data sets can also be further combined with large-scale binary interactions based on yeast two-hybrid and cofractionation MS to build a more complete interaction map (2, 74, 119).

Another emerging research interest is to couple cell type–specific PL-MS with single-cell RNA-sequencing and proteomics. A quantitative atlas of the *Arabidopsis* proteome has been achieved at the tissue level (75) but not at cell-type nor single-cell level. While single-cell proteomics is still challenging (88, 89), it will gain momentum soon. With a joint effort of the community, generating such a wealth of data at different scales with a myriad of approaches will pave the way toward creating a plant cell atlas in the future (97).

## Supplementary Material

Supplemental Table 1

## Figures and Tables

**Table 1:** Comparison of the enrichment of biotin-labeled proteins at protein and peptide levels in plants

Enrichment	Biotin concentration	Evidence	Sensitivity	Reagents/equipment	Duration	Additional information
At protein level	Only allows a lower concentration of biotin treatment (≤50 μM)	Indirect evidence	More sensitive due to more abundance of nonbiotinylated peptides than of biotinylated peptides	Size-exclusion column (e.g., PD-10) to remove free biotin	4–5 days, including 2–3 days to perform enrichment (done by user) and 2 days on-bead digestion (done by MS facility)	Not applicable
At peptide level	Allows a higher concentration of biotin treatment, as biotin can be efficiently removed during protein precipitation	Direct and highly confident evidence	If biotinylated peptides are efficiently enriched, it can produce similar results due to reduced sample complexity for MS acquisition	Trypsin and desalting ZipTips for digestion and desalting and SpeedVac for drying peptides	4–5 days (done by user)	Provides topology information and surface-exposed region of proteins.

**Table 2:** Comparison of quantification methods used in proximity-labeling mass spectrometry

Labeling	Applied plant species	Multiplexing samples	Growth duration	Special reagents/kit needed	Fractionation to increase coverage	Quantification	Most used software for data analysis
Label-free quantification (LFQ)	All species	None	No restriction	No	No	MS1 (precursor), ratio of summed peptide signals	Maxquant/Perseus (24, 121, 122)
Multiplexed TMT isobaric labeling	All species	Up to 18	No restriction	TMT isobaric tagging kit	Yes	Ratios from MS2 or MS3 fragments	Maxquant/Perseus (24, 121, 122), Protein Discoverer, Protein Prospector (109)
^15^N metabolic labeling	*Arabidopsis, Chlamydomonas*	2	≥14 days, unless seeds are metabolic labeled for one generation	^15^N salt (K^15^NO_3;_^15^NH4^15^NO3)	Yes	MS1 (precursor), median of ratios of peptides	Protein Prospector (109), pFIND (66)

## SUMMARY POINTS

1. Proximity-labeling mass spectrometry (PL-MS) is a powerful tool used to dissect protein–protein interactions (PPIs), subcellular proteomes, and cell type–specific proteomes.
2. Engineered high-activity TurboID has extended its applications to multiple species and to answer more biological questions.
3. Modular designs of PL-MS have enabled the mapping of conditional PPIs and hard-to-purify subcellular structures and have improved the versatility of PL applications.
4. Coupling CRISPR with PL has opened a new avenue for probing the landscape of defined genome loci and for mapping RNA-binding protein complexes.
5. PL-MS can be combined with posttranslational modification studies to dissect regulatory mechanisms in the future.
6. PL-MS can be coupled with cross-linking MS and single-cell proteomics to build a plant cell atlas that will be crucial to the plant community.

## Abbreviations

CRISPR: clustered regularly interspaced short palindromic repeats
LC-MS/MS: liquid chromatography–coupled tandem mass spectrometry
DR5: auxin-responsive promoter
TCSn: two-component signaling sensor/cytokinin-responsive promoter
XIST: X-inactive-specific transcript
λN: a 22–amino acid RNA-binding domain of λ bacteriophage antiterminator protein N
BoxB: λN’s specific 19-nucleotide binding site
MS2: a stem-loop structure from the phage genome
MCP: MS2 bacteriophage coat protein that binds to the MS2 stem loop
MS1: precursor ion scan or the first stage of tandem mass spectrometry
MS/MS (MS2): fragment ion scan after fragmenting the isolated precursor ions from the MS1 level, or the second stage of tandem mass spectrometry
MS/MS/MS (MS3): fragment ion scan after fragmenting the isolated ions from the MS2 level, or the third stage of tandem mass spectrometry
LOESS-R: locally weighted scatterplot-smoothing regression
