# Supplementary material for: Proximity Labeling in Plants
Source: Annu Rev Plant Biol. Author manuscript; Available in PMC 2023 Oct 14. (PMC10576617; doi:10.1146/annurev-arplant-070522-052132)
Supplement: Supplemental Table 1 [file NIHMS1934875-supplement-Supplemental_Table_1.pdf]

Supplemental Material: Annu. Rev. Plant Biol. 2023. 74.
https://doi.org/10.1146/annurev-arplant-070522-052132
Proximity Labeling in Plants
Xu, Shrestha, Karunadasa, and Xie

Supplemental Table 1: Comparison of different biotin ligases used in the PL-MS applications.

Enzyme name	Labeling time based on MS experiment /Conditions (speed of labeling)	Organelles/subcellular structures demonstrated in other species	Organelles/subcellular structures demonstrated in plants	Plant species tested
BioID	18-24h, Optimum temperature 37℃	Membrane-bound organelles: Nucleus (8) and its sub-nuclear compartment and domains, including nuclear pore complex (16,26,35),nuclear lamina (12,16), nucleolus (4,16), nucleoplasm (16); mitochondria including matrix (9,15,16,22); Golgi (6,16) Endoplasmic Reticulum lumen (12,16), peroxisome (16), lysosome (16)<br><br>Membrane: ER membrane (12,16), peroxisome membrane (20), mitochondria inner (16) and outer membrane (16,22), cell membrane (16,40), Golgi membrane (16), nuclear membrane (16)<br><br>Other subcellular structures: centrosome (10,14,16); centrosome-cilium interface (18); cytosol (3,16) and cytoskeleton (16)<br><br>Junctions: cell junctions (16-18,42,43)	Membrane-bound organelles: Nucleus (31,34)<br><br>Membrane: Cell membrane (1,11,24)<br><br>Other subcellular structure: cytosol (1,13,34)	*Nicotiana benthamiana* (1,11,13)<br>(33); *Arabidopsis thaliana* (24,49)<br>*Oryza sativa L.* (rice protoplasts) (31)<br>*Solanum lycopersicum* (tomato hairy root culture) (1)
TurboID	≥ 10 min, 30-37℃ temperature optimum, works well at 20-25 ℃	Membrane-bound organelles: nucleus (3) and its sub-nuclear compartment and domains, including nuclear lamina (35,39) and nuclear pore complex (35); mitochondria including matrix (3); ER lumen (3,35); exosome (30)<br><br>Membrane: ER membrane (3).	Membrane-bound organelles: Nucleus and its-subnuclear compartment and domains (34), close to the nuclear pore region & nucleus (44)<br><br>Membrane: Cell membrane (1)	*Nicotiana benthamiana* (1,34,48,49), *Arabidopsis thaliana* (28,34,44); Arabidopsis cell cultures (1); *Solanum lycopersicum* (tomato hairy root culture) (1), *Zea Mays* and Citrus (our

		Other subcellular structure:<br>Cytosol (3), centrosome (19)	Other subcellular structure:<br>Cytosol (28,34)	unpublished data)
MiniTurboID	≥ 10 min, 30℃ temperature optimum, works well at 20-25 ℃	Membrane-bound organelles:<br>Nucleus (3) and its sub-nuclear compartment and domains, including nuclear lamina (39), mitochondrial matrix (3), ER lumen (3)<br><br>Membrane: ER membrane (3)<br><br>Other subcellular structure: Cytosol (3)	Membrane-bound organelles:<br>Nucleus (34)<br><br>Membrane: Plasma membrane (36)<br><br>Other subcellular structure:<br>Cytosol (34)	*Nicotiana benthamiana* (1,34), *Arabidopsis thaliana* (1,34), *Solanum lycopersicum* (Tomato hairy root culture) (1), *Marchantia polymorpha* (liverwort) (36)
BioID2	16-24h,<br>50℃ temperature optimum, works well at 37℃, lower activity at 22℃.	Membrane-bound organelles:<br>Golgi (32)<br><br>Membrane:<br>Nuclear inner membrane (27), ER membrane (2), Mitochondrial outer membrane (7), Mitochondrial inner membrane (5)<br><br>Other subcellular structure:<br>Cytoplasm (3,27), Nuclear pore complex (27), Mitochondrial intermembrane space (47)	Membrane-bound organelles:<br>nucleus and its sub-nuclear compartment and domains, including nuclear pore complex (41)<br><br>Membrane:<br>Cell membrane (1), Inner nuclear membrane (21,41) and outer nuclear membrane (21,41)<br><br>Other subcellular structure:<br>Cytosol (1)	*Nicotiana benthamiana* (1,49), *Arabidopsis thaliana* (21,41), *Solanum lycopersicum* (tomato hairy root culture) (1)
microID	n.a.	Cytosol (29)	n.a.	Not tested in plants
ultraID	≥ 10 min	Other subcellular structure:<br>Cytosol (29)<br><br>Membrane:<br>Golgi vesicle membrane (29), cell membrane (40)	n.a.	Not tested in plants
microID2	3h labeling time	Membrane-bound organelles:<br>Nucleus (23), Mitochondria (23)	n.a.	Not tested in plants

		Membrane: cell membrane (23)<br><br>Other subcellular structure: Cytoplasm (23)		
lbMicroID2	n.a.	Membrane: cell membrane (23)<br><br>Other subcellular structure: Cytoplasm (23)	n.a.	Not tested in plants
AirID	3-8h, Activity has been tested in the temperature range of 16-37℃	Membrane-bound organelles: Nucleus (25,45)<br><br>Other subcellular structure: Cytosol (25,45)	n.a.	Not tested in plants
BASU	30 min -18 h	Membrane-bound organelles: Nucleus (38,46)<br><br>Other subcellular structure: cytosol (3,37,38,46)	n.a.	Not tested in plants

REFERENCE

1. Arora D, Abel NB, Liu C, Van Damme P, Yperman K, et al. 2020. Establishment of Proximity-Dependent Biotinylation Approaches in Different Plant Model Systems. *Plant Cell* 32:3388-407
2. Bagchi P, Torres M, Qi L, Tsai B. 2020. Selective EMC subunits act as molecular tethers of intracellular organelles exploited during viral entry. *Nat Commun* 11:1127
3. Branon TC, Bosch JA, Sanchez AD, Udeshi ND, Svinkina T, et al. 2018. Efficient proximity labeling in living cells and organisms with TurboID. *Nat. Biotechnol.* 36:880-7
4. Brecht RM, Liu CC, Beilinson HA, Khitun A, Slavoff SA, Schatz DG. 2020. Nucleolar localization of RAG1 modulates V(D)J recombination activity. *Proceedings of the National Academy of Sciences* 117:4300-9
5. Callegari S, Muller T, Schulz C, Lenz C, Jans DC, et al. 2019. A MICOS-TIM22 Association Promotes Carrier Import into Human Mitochondria. *J Mol Biol* 431:2835-51
6. Chan CJ, Le R, Burns K, Ahmed K, Coyaud E, et al. 2019. BioID Performed on Golgi Enriched Fractions Identify C10orf76 as a GBF1 Binding Protein Essential for Golgi Maintenance and Secretion. *Mol. Cell. Proteomics* 18:2285-97
7. Chen Z, Lei C, Wang C, Li N, Srivastava M, et al. 2019. Global phosphoproteomic analysis reveals ARMC10 as an AMPK substrate that regulates mitochondrial dynamics. *Nat Commun* 10:104
8. Chojnowski A, Ong PF, Wong ES, Lim JS, Mutalif RA, et al. 2015. Progerin reduces LAP2alpha-telomere association in Hutchinson-Gilford progeria. *Elife* 4
9. Cole A, Wang Z, Coyaud E, Voisin V, Gronda M, et al. 2015. Inhibition of the Mitochondrial Protease ClpP as a Therapeutic Strategy for Human Acute Myeloid Leukemia. *Cancer Cell* 27:864-76
10. Comartin D, Gupta GD, Fussner E, Coyaud E, Hasegan M, et al. 2013. CEP120 and SPICE1 cooperate with CPAP in centriole elongation. *Curr Biol* 23:1360-6
11. Conlan B, Stoll T, Gorman JJ, Saur I, Rathjen JP. 2018. Development of a Rapid in planta BioID System as a Probe for Plasma Membrane-Associated Immunity Proteins. *Front Plant Sci* 9:1882
12. Cross SH, McKie L, Hurd TW, Riley S, Wills J, et al. 2020. The nanophthalmos protein TMEM98 inhibits MYRF self-cleavage and is required for eye size specification. *PLoS Genet.* 16:e1008583
13. Das PP, Macharia MW, Lin Q, Wong S-M. 2019. In planta proximity-dependent biotin identification (BioID) identifies a TMV replication co-chaperone NbSGT1 in the vicinity of 126 kDa replicase. *Journal of Proteomics* 204:103402
14. Firat-Karalar EN, Rauniyar N, Yates JR, 3rd, Stearns T. 2014. Proximity interactions among centrosome components identify regulators of centriole duplication. *Curr Biol* 24:664-70
15. Gillingham AK, Bertram J, Begum F, Munro S. 2019. In vivo identification of GTPase interactors by mitochondrial relocalization and proximity biotinylation. *Elife* 8
16. Go CD, Knight JDR, Rajasekharan A, Rathod B, Hesketh GG, et al. 2021. A proximity-dependent biotinylation map of a human cell. *Nature* 595:120-4

17. Guo Z, Neilson LJ, Zhong H, Murray PS, Zanivan S, Zaidel-Bar R. 2014. E-cadherin interactome complexity and robustness resolved by quantitative proteomics. *Sci Signal* 7:rs7
18. Gupta GD, Coyaud E, Goncalves J, Mojarad BA, Liu Y, et al. 2015. A Dynamic Protein Interaction Landscape of the Human Centrosome-Cilium Interface. *Cell* 163:1484-99
19. Holzer E, Rumpf-Kienzl C, Falk S, Dammermann A. 2022. A modified TurboID approach identifies tissue-specific centriolar components in C. elegans. *PLOS Genetics* 18:e1010150
20. Hua R, Cheng D, Coyaud E, Freeman S, Di Pietro E, et al. 2017. VAPs and ACBD5 tether peroxisomes to the ER for peroxisome maintenance and lipid homeostasis. *J Cell Biol* 216:367-77
21. Huang A, Tang Y, Shi X, Jia M, Zhu J, et al. 2020. Proximity labeling proteomics reveals critical regulators for inner nuclear membrane protein degradation in plants. *Nat. Commun.* 11:3284
22. Janer A, Prudent J, Paupe V, Fahiminiya S, Majewski J, et al. 2016. SLC25A46 is required for mitochondrial lipid homeostasis and cristae maintenance and is responsible for Leigh syndrome. *EMBO Mol. Med.* 8:1019-38
23. Johnson BS, Chafin L, Farkas D, Adair J, Elhance A, et al. 2022. MicroID2: A Novel Biotin Ligase Enables Rapid Proximity-Dependent Proteomics. *Mol Cell Proteomics* 21:100256
24. Khan M, Youn JY, Gingras AC, Subramaniam R, Desveaux D. 2018. In planta proximity dependent biotin identification (BioID). *Sci Rep* 8:9212
25. Kido K, Yamanaka S, Nakano S, Motani K, Shinohara S, et al. 2020. AirID, a novel proximity biotinylation enzyme, for analysis of protein-protein interactions. *Elife* 9
26. Kim DI, Birendra KC, Zhu W, Motamedchaboki K, Doye V, Roux KJ. 2014. Probing nuclear pore complex architecture with proximity-dependent biotinylation. *Proc. Natl. Acad. Sci. U. S. A.* 111:E2453-61
27. Kim DI, Jensen SC, Noble KA, Kc B, Roux KH, et al. 2016. An improved smaller biotin ligase for BioID proximity labeling. *Mol. Biol. Cell* 27:1188-96
28. Kim T-W, Park CH, Hsu C-C, Zhu J-Y, Hsiao Y, et al. 2019. Application of TurboID-mediated proximity labeling for mapping a GSK3 kinase signaling network in Arabidopsis. *bioRxiv*:636324
29. Kubitz L, Bitsch S, Zhao X, Schmitt K, Deweid L, et al. 2022. Engineering of ultraID, a compact and hyperactive enzyme for proximity-dependent biotinylation in living cells. *Commun Biol* 5:657
30. Larochelle M, Bergeron D, Arcand B, Bachand F. 2019. Proximity-dependent biotinylation mediated by TurboID to identify protein-protein interaction networks in yeast. *J. Cell Sci.* 132
31. Lin Q, Zhou Z, Luo W, Fang M, Li M, Li H. 2017. Screening of Proximal and Interacting Proteins in Rice Protoplasts by Proximity-Dependent Biotinylation. *Front. Plant Sci.* 8:749
32. Liu L, Doray B, Kornfeld S. 2018. Recycling of Golgi glycosyltransferases requires direct binding to coatomer. *Proc Natl Acad Sci U S A* 115:8984-9
33. Macharia MW, Tan WYZ, Das PP, Naqvi NI, Wong S-M. 2019. Proximity-dependent biotinylation screening identifies NbHYPK as a novel interacting partner of ATG8 in plants. *BMC Plant Biol.* 19:326
34. Mair A, Xu S-L, Branon TC, Ting AY, Bergmann DC. 2019. Proximity labeling of protein complexes and cell-type-specific organellar proteomes in enabled by TurboID. *Elife* 8

35. May DG, Scott KL, Campos AR, Roux KJ. 2020. Comparative Application of BioID and TurboID for Protein-Proximity Biotinylation. *Cells* 9
36. Melkonian K, Stolze SC, Harzen A, Nakagami H. 2022. miniTurbo-based interactomics of two plasma membrane-localized SNARE proteins in Marchantia polymorpha. *New Phytol.* 235:786-800
37. Mirza AN, McKellar SA, Urman NM, Brown AS, Hollmig T, et al. 2019. LAP2 Proteins Chaperone GLI1 Movement between the Lamina and Chromatin to Regulate Transcription. *Cell* 176:198-212.e15
38. Ramanathan M, Majzoub K, Rao DS, Neela PH, Zarnegar BJ, et al. 2018. RNA-protein interaction detection in living cells. *Nat Methods* 15:207-12
39. Rosenthal SM, Misra T, Abdouni H, Branon TC, Ting AY, et al. 2021. A Toolbox for Efficient Proximity-Dependent Biotinylation in Zebrafish Embryos. *Mol. Cell. Proteomics* 20:100128
40. Salokas K, Liu X, Öhman T, Chowdhury I, Gawriyski L, et al. 2022. Physical and functional interactome atlas of human receptor tyrosine kinases. *EMBO reports* 23
41. Tang Y, Huang A, Gu Y. 2020. Global profiling of plant nuclear membrane proteome in Arabidopsis. *Nat Plants* 6:838-47
42. Ueda S, Blee AM, Macway KG, Renner DJ, Yamada S. 2015. Force dependent biotinylation of myosin IIA by α-catenin tagged with a promiscuous biotin ligase. *PLoS One* 10:e0122886
43. Van Itallie CM, Tietgens AJ, Aponte A, Fredriksson K, Fanning AS, et al. 2014. Biotin ligase tagging identifies proteins proximal to E-cadherin, including lipoma preferred partner, a regulator of epithelial cell-cell and cell-substrate adhesion. *J Cell Sci* 127:885-95
44. Xu F, Jia M, Li X, Tang Y, Jiang K, et al. 2021. Exportin-4 coordinates nuclear shuttling of TOPLESS family transcription corepressors to regulate plant immunity. *Plant Cell* 33:697-713
45. Yamanaka S, Horiuchi Y, Matsuoka S, Kido K, Nishino K, et al. 2022. A proximity biotinylation-based approach to identify protein-E3 ligase interactions induced by PROTACs and molecular glues. *Nat. Commun.* 13:183
46. Yi W, Li J, Zhu X, Wang X, Fan L, et al. 2020. CRISPR-assisted detection of RNA–protein interactions in living cells. *Nature Methods* 17:685-8
47. Yoshinaka T, Kosako H, Yoshizumi T, Furukawa R, Hirano Y, et al. 2019. Structural Basis of Mitochondrial Scaffolds by Prohibitin Complexes: Insight into a Role of the Coiled-Coil Region. *iScience* 19:1065-78
48. Zhang Y, Li Y, Yang X, Wen Z, Nagalakshmi U, Dinesh-Kumar SP. 2020. TurboID-Based Proximity Labeling for In Planta Identification of Protein-Protein Interaction Networks. *J. Vis. Exp.*
49. Zhang Y, Song G, Lal NK, Nagalakshmi U, Li Y, et al. 2019. TurboID-based proximity labeling reveals that UBR7 is a regulator of N NLR immune receptor-mediated immunity. *Nature Communications* 10
